# Dietary Patterns Associated with Body-Composition Phenotype in a Middle-Aged and Elderly Population: A Population-Based Cross-Sectional Study

**DOI:** 10.3390/nu16213583

**Published:** 2024-10-22

**Authors:** Jong Eun Park, Narae Yang, Kirang Kim

**Affiliations:** 1 Institute of Health & Science Convergence, Chungbuk National University, Cheongju 28644, Republic of Korea; je.park0525@gmail.com; 2 Department of Food Science and Nutrition, Dankook University, Cheonan 31116, Republic of Korea; 2nalgae@gmail.com

**Keywords:** sarcopenia, low muscle mass, sarcopenic obesity, dietary pattern, healthy diet, alcohol

## Abstract

Background/Objectives: There is currently limited understanding of the intricate dynamics between fat and muscle mass and the potential effect of dietary patterns. This study aimed to investigate the effects of overall dietary patterns on four body composition phenotypes associated with central obesity and low muscle mass in the middle-aged and elderly population of Korea. Methods: We examined data from 6120 adults aged ≥ 50 years, collected during the 4th (2008–2009) and 5th (2010–2011) Korea National Health and Nutrition Examination Survey. Participants were classified into four groups according to their combined status of central obesity and low muscle mass: healthy control, central obesity, low muscle mass, and sarcopenic obesity. Using factor analysis on the twenty-two pre-defined food groups, we identified three distinct dietary patterns: the “Healthy dietary pattern”, “Convenience-based dietary pattern”, and “Alcohol and side dish pattern”. Multivariate analysis included potential confounders such as age, sex, place of residence, marital status, educational level, occupation, household income, and physical activity level. Results: Higher scores in the “Healthy dietary pattern” were associated with lower odds of sarcopenic obesity (OR = 0.61, 95% CI = 0.40–0.92, p for trend = 0.018). For the “Convenience-based dietary pattern”, individuals in the third tertile of the dietary pattern score showed a marginal association with low muscle mass (OR = 1.18, 95% CI = 0.99–1.41, p for trend = 0.056). The “Alcohol and side dish pattern” was linked to higher odds of central obesity (OR = 1.22, 95% CI = 1.03–1.44, p for trend = 0.016) and low muscle mass (OR = 1.21, 95% CI = 1.01–1.45, p for trend = 0.043). Conclusions: The drinking practice of consuming foods high in saturated fats and salt alongside alcoholic drinks may negatively affect fat accumulation and muscle composition among older adults. Adopting a healthy eating pattern characterized by grains, vegetables, legumes, fruits, fish, and seaweed could be notably advantageous for promoting and maintaining healthy body composition and optimal skeletal muscle health.

## 1. Introduction

Aging is widely recognized for its detrimental effects on various components of the musculoskeletal system, encompassing joints, bones, and muscles, as well as its broader impact on multiple body systems [1]. Moreover, aging often leads to an overall increase in body fat, particularly visceral fat, which tends to accumulate in the abdominal region [2]. These changes typically begin in mid-adulthood and continue to progress gradually over time. Research suggests that muscle mass decreases at an approximate rate of 3–8% per decade after the age of 30, with this rate of decline accelerating further after the age of 60 [3,4]. These alterations in body composition associated with aging may be closely related to reduced mobility, diminished functional capacity [1], and a heightened susceptibility to conditions such as cardiovascular diseases, diabetes, and metabolic syndrome [2].

The prevalence of sarcopenia examined using handgrip strength data from the Korea National Health and Nutrition Examination Survey (KNHANES) (2014–2017) showed that the proportion in elderly men increased from 22.6% in 2014 to 29.3% in 2017 and the proportion in elderly women increased from 19.3% in 2014 to 30.1% in 2017 [5]. In addition, obesity prevalence has steadily increased over the past 11 years between 2009 and 2019 in all ages; from 29.7% in 2009 to 36.3% in 2019 for the total population. Specifically, the prevalence of abdominal obesity increased between 2009 (19.0%) and 2019 (23.9%) and the increase in women was more moderate than in men. It also increases with age, showing a higher prevalence among the middle-aged and older population compared to the younger population [6]. Therefore, the double burden of these outcomes is a major concern within the aging population.

Aging-related increases in body fat and declines in skeletal muscle mass are exacerbated by nutritional imbalances and insufficient exercise, which has been highlighted in recent studies on sarcopenic obesity and sarcopenia, and obesity [7,8]. Specifically, an extensive number of studies have used a single-nutrient or -food approach to examine the relationship between diet and body-composition phenotypes. However, it is difficult to isolate the impact of an individual dietary component while considering the interactions among various components within a complete diet and their effects on health outcomes [9,10]. Therefore, dietary patterns that offer a more comprehensive view on how nutrient and bioactive components interact within the context of an entire diet have recently been used to examine dietary influence on aging muscle and adiposity [11,12,13,14]. In particular, studies exploring the relationship between diet and sarcopenia emphasize common principles, such as a high intake of whole grains, vegetables, and fruits, along with a lower consumption of highly processed foods [15].

It is important to conduct a proper evaluation and early intervention for nutrition to manage body composition in middle-aged individuals and older adults to prevent the development of obesity or sarcopenia. However, the intricate relationship between fat and muscle mass, as well as the potential effect of dietary patterns, is not yet fully understood. Therefore, the objective of this study was to investigate the effect of dietary patterns on various body composition phenotypes, including central obesity, low muscle mass, and sarcopenic obesity, among the middle-aged and older adults, using data from the representative Korean population provided by the KNHANES.

## 2. Materials and Methods

### 2.1. Data Source and Study Population

**Figure 1:**
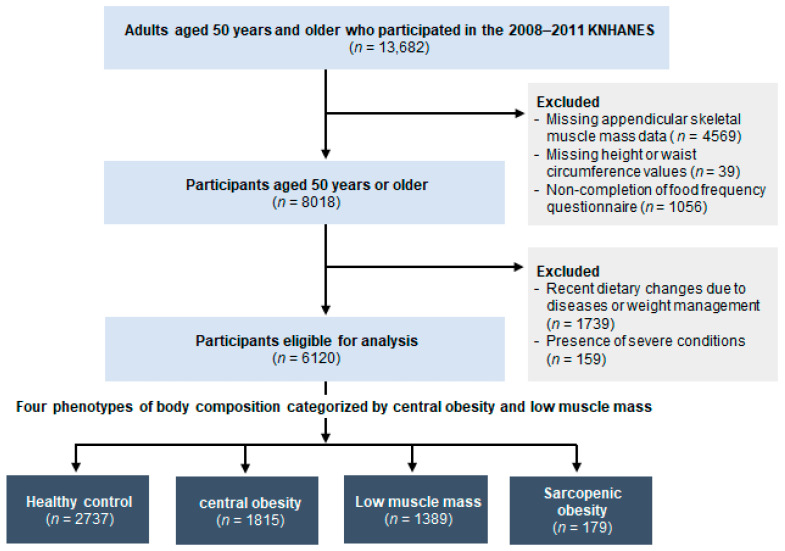
Flow diagram for study participants. KNHANES, Korea National Health and Nutrition Examination Survey.

This population-based cross-sectional study utilized data from the 4th (2008–2009) and 5th (2010–2011) KNHANES, which is an annual national health survey that evaluates the health status and health-related behaviors of the Korean population through health interviews, physical examinations, and nutrition surveys [16]. Of the 13,682 adults aged ≥ 50 years who participated in the 2008–2011 KNHANES, a total of 8018 individuals who underwent the simple food frequency questionnaire (FFQ) and anthropometric assessment, including dual-energy X-ray absorptiometry (DXA) screening, were included in this study. Subsequently, participants meeting any of the following exclusion criteria were removed: (i) recent dietary changes for managing diseases or weight management (*n* = 1739); and (ii) presence of severe conditions like cancer, renal failure, and liver cirrhosis (*n* = 159). Ultimately, 1898 participants were excluded, resulting in 6120 participants who were eligible for the final analysis (Figure 1).

### 2.2. Definition of Low Muscle Mass and Central Obesity

Muscle mass was assessed using DXA (Discovery-W fan-beam densitometer, Hologic, Inc., Marlborough, MA, USA). Appendicular skeletal muscle mass (ASM) was determined by summing the muscle mass of the upper and lower limbs, excluding fat and bone tissue. Subsequently, the appendicular skeletal muscle mass index (ASMI) was computed by dividing the ASM by the square of the individual’s height (kg/m^2^). Low muscle mass (LMM), one of the diagnostic criteria for sarcopenia, was identified as an ASMI of <5.4 kg/m^2^ in women and <7.0 kg/m^2^ in men [17]. This specific cutoff was suggested by the Asian Working Group for Sarcopenia (AWGS), considering the ethnic variations in skeletal muscle mass among older individuals in Asian populations.

Anthropometric measurements, including height, weight, and waist circumference, were taken by trained examiners using standardized methods. Height and weight were measured with light clothing and without shoes. Waist circumference was measured at the midpoint between the lower rib cage margin and the top of the iliac crest, with a precision of 0.1 cm. The criteria of the Korean Society for the Study of Obesity were employed to define central obesity (CO), which entailed a waist circumference of ≥90 cm in men and ≥85 cm in women [18]. Sarcopenic obesity (SO) was characterized by the concurrent presence of both LMM and CO. The participants were categorized into four groups based on their combined status of LMM and CO: (i) healthy control (HC) (non-LMM and non-CO), (ii) CO (non-LMM and CO), (iii) LMM (non-CO and LMM), and (iv) SO (LMM and CO).

### 2.3. Assessment of Dietary Factors

Dietary intake information was collected using a non-quantitative, frequency-based FFQ (without portion-size questions). The FFQ includes sixty-three food and beverage items representing the most frequently consumed foods and nutrients among the Korean population [19]. It has been widely used in numerous diet-disease studies in Korea, supporting its validity [20,21]. Participants were required to indicate how often they consumed each food item throughout the previous year, and these frequencies were classified into the following ten categories: not consumed or consumed very infrequently; 6–11 times per year; 1 time per month; 2–3 times per month; 1 time per week; 2–3 times per week; 4–6 times per week; 1 time per day; 2 times per day; and 3 times per day. For dietary pattern analysis, the midpoint of each frequency category was converted to a weekly intake frequency. For instance, if a participant consumed rice three times daily, their rice intake frequency was 21 times per week. These sixty-three food items were further categorized into twenty-two food groups based on nutrient composition and food preparation methods. Foods that did not fit into a specific group or unique foods (e.g., rice, eggs, mushrooms, and soda) were maintained as individual categories. The ensuing food groups were defined as follows: rice; other gains; noodles; bread, sweets, and confectionery; rice cakes; legumes and legume products; potatoes and sweet potatoes; meats (beef, pork, and chicken); eggs; processed meats; fish and seafood; salted fish and seafood; vegetables; mushrooms; seaweed; fruits; dairy products; soda; coffee and tea; alcoholic beverages; fast foods (hamburgers and pizza); and fried foods (Supplementary Table S1). Dietary patterns were generated using factor analysis based on twenty-two pre-defined food groups.

### 2.4. Other Variables

Levels of physical activity, comorbidities, and sociodemographic characteristics, including age, sex, place of residence (urban and rural areas), marital status (married, separated/divorced/widowed, or never married), educational level (elementary school graduate or less, middle school graduate, high school graduate, or college graduate or more), occupation (white-collar worker, pink-collar worker, blue-collar worker, or unemployed), and household income (quartiles in each period), were considered as covariates. Household income level was estimated as equivalized total household income by dividing the total household income by the square root of the number of people in the household, and then divided into quartiles for each period. Physical activity was assessed using the Korean version of the International Physical Activity Questionnaire (IPAQ) short form, which inquired about the frequency (number of days) and duration (minutes that each activity lasted) of moderate- or vigorous-intensity activities per week. Participants were categorized into three groups based on their combined minutes of moderate- and vigorous-intensity activities: no physical activity, insufficient physical activity level, and recommended physical activity level. Engaging in vigorous-intensity physical activity for a minimum of 75 min per week, moderate-intensity physical activity for at least 150 min per week, or a combination of both moderate- and vigorous-intensity physical activities totaling at least 75 min per week was categorized as meeting the recommended physical activity level [22]. When merging moderate- and vigorous-intensity activities, 2 min of moderate-intensity activity was considered equivalent to 1 min of vigorous-intensity activity. Hypertension and diabetes mellitus were considered comorbidities. Hypertension was defined as a self-reported history of hypertension, current use of antihypertensive drugs, systolic blood pressure of ≥140 mmHg, or diastolic blood pressure of ≥90 mmHg. Diabetes mellitus was defined as a self-reported history of diabetes mellitus, current use of insulin or oral hypoglycemic agents, or a fasting glucose level of ≥126 mg/dL.

### 2.5. Statistical Analysis

We used a chi-square test and one-way analysis of variance (ANOVA) to compare the characteristics of participants based on their combined status of LMM and CO.

Dietary patterns and their factor scores were generated by factor analysis, conducted using the PROC FACTOR procedure. The analysis utilized twenty-two pre-defined food items as independent variables. The factors were rotated with an orthogonal transformation using a varimax rotation to achieve a simpler structure with greater interpretability. Varimax rotation is commonly used as it maximizes the variance of the squared loadings across factors, thereby simplifying the structure and emphasizing the most significant variables contributing to each factor. The number of factors to be retained was determined based on several criteria, including a minimum eigenvalue of 1.0, examination of the scree plot, and the interpretability of the factors. Using these criteria, three factors were identified, each representing a distinct dietary pattern within the study population. Each food item was assigned a factor loading, which quantified its contribution to the respective factor. Food items with an absolute factor loading of 0.40 or greater were considered to make a significant contribution to the dietary pattern and were subsequently interpreted in defining the dietary patterns. Factor scores for each individual were calculated for each of the three dietary patterns. These scores represented the extent to which each individual’s diet aligned with the identified dietary patterns. For subsequent analysis, the factor scores were categorized into tertiles, thereby classifying the study population into three groups based on adherence to each dietary pattern.

Multinomial logistic regression was used to assess the associations of dietary pattern tertiles with CO, LMM, and SO. We estimated odds ratios (ORs) and 95% confidence intervals (CIs) for CO, LMM, and SO, adjusted for age, sex, place of residence, marital status, educational level, occupation, household income, and physical activity level. All analyses were conducted using SAS software version 9.4 (SAS Institute Inc., Cary, NC, USA), and a two-tailed *p* < 0.05 was considered statistically significant.

## 3. Results

The participants’ general characteristics across the four phenotypes of waist circumference and appendicular muscle mass are summarized in Table 1. Among the participants, 44.7% were classified under the HC group. The CO group comprised 29.7%, the LMM group 22.7%, and the SO group 2.9%. Compared to the other groups, the proportion of men was significantly higher in the LMM group (55.7%), while the proportion of women was significantly higher in the CO group (63.9%) (*p* < 0.001). The SO group was older, less educated, at lower income levels, more likely to be separated, divorced, or widowed, and unemployed compared to the other groups (all *p* < 0.01). The proportion of individuals meeting the recommended level of physical activity was lowest in the SO group and highest in the HC group (*p* < 0.001). The proportion of individuals with hypertension or diabetes mellitus was lowest in the HC group and highest in the SO group (both *p* < 0.001).

Three dietary patterns were identified: “healthy dietary pattern”, “convenience-based dietary pattern”, and “alcohol and side dish pattern” (Table 2). The first pattern exhibited the highest factor loadings for grains, legumes and legume products, potatoes and sweet potatoes, fish and seafood, vegetables, mushrooms, seaweed, and fruits. This pattern, which is primarily plant-based and includes a high intake of legumes, fish, and seafood as protein sources, was labeled the “healthy dietary pattern” and closely aligns with the traditional Korean diet. The second pattern exhibited the highest factor loadings for bread, sweets, and confectionery, rice cake, dairy products, and fast food (hamburger and pizza), while showing negative loadings for rice. This pattern, characterized by the frequent consumption of processed and fast foods, often chosen for their convenience, was labeled the “convenience-based dietary pattern”. The third pattern, labeled the “alcohol and side dish pattern” was characterized by a high intake of noodles, meat (beef, pork, and chicken), salted fish and seafood, and alcohol. This pattern reflects dietary habits associated with social drinking, where alcohol is consumed alongside side dishes such as meat and salty preserved foods.

As shown in Table 3, the score distribution of the “healthy dietary pattern” and the “alcohol and side dish pattern” differed across the four phenotypes. The HC group was more likely to be in the highest tertile of the “healthy dietary pattern”, while the SO group was more likely to be in the lowest tertile (*p* = 0.002). The LMM group was more likely to be in the highest tertile of the “alcohol and side dish pattern” (*p* = 0.005).

Table 4 shows the results of multinomial logistic regression models investigating the relationship between dietary patterns and four body composition phenotypes associated with CO and LMM (reference group was the HC). Participants with high adherence (highest tertile) to the “healthy dietary pattern” were less likely to have sarcopenic obesity compared to those in the lowest tertile, after adjusting for covariates (OR = 0.61, 95% CI = 0.40–0.92, p for trend = 0.018). For the “convenience-based dietary pattern”, individuals in the highest tertile of adherence were marginally associated with having LMM, after adjusting for covariates (OR = 1.18, 95% CI = 0.99–1.41, p for trend = 0.056). Additionally, those in the second tertile of adherence were associated with CO (OR = 1.21, 95% CI = 1.04–1.41). The “alcohol and side dish pattern” was related to higher odds of CO (OR = 1.22, 95% CI = 1.03–1.44, p for trend = 0.016), and LMM (OR = 1.21, 95% CI = 1.01–1.45, p for trend = 0.043). 

## 4. Discussion

Current research investigating optimal dietary patterns for maintaining healthy body composition and preventing skeletal muscle mass loss remains insufficient. Moreover, considering the simultaneous high accumulation of fat mass and low skeletal muscle mass with aging, the evidence on the effect of dietary patterns depending on body composition phenotype is limited. The objective of this study was to investigate whether the effect of dietary patterns is different by body composition phenotype among the middle-aged and older population. This study considered three dietary patterns: healthy dietary pattern, convenience-based dietary pattern, and alcohol and side dish pattern. In terms of the relationship with body composition phenotype, the risk of obesity was positively related to the alcohol and side dish pattern, the risk of LMM was positively related to both the convenience-based dietary pattern and the alcohol and side dish pattern, and the risk of SO was negatively related to the healthy dietary pattern.

While many studies have examined the relationship between specific diets and sarcopenia, research specifically focusing on dietary patterns associated with SO has been limited. In a study investigating the association between dietary patterns and SO among middle-aged women, adherence to the ‘unbalanced diet pattern’—characterized by increased consumption of alcohol and ultra-processed foods such as noodles, fish cakes, and soft drinks, alongside decreased intake of grains and kimchi—was associated with a higher odds for SO [23]. Conversely, adherence to the DASH diet was linked to a reduced risk of SO in overweight and obese women [24]. Additionally, adherence to the Mediterranean diet has been shown to not only affect muscle mass but also impact BMI, waist circumference, and body fat percentage in individuals with obesity [25].

In our study, the healthy dietary pattern, characterized by a diverse intake of plant-based foods and seafood, reflecting the grain-based diet typical of the traditional Korean diet, was associated with a 39% lower probability of SO, suggesting a potential preventive effect. This dietary pattern aligns closely with several key components of the Mediterranean diet, which is widely recognized for its health-promoting properties. Specifically, this pattern may contribute to improved metabolic health and the prevention of muscle degradation by emphasizing nutrient-dense foods, particularly vegetables, mushrooms, and fruits that are rich in vitamins, minerals, and fiber, as well as fish high in protein and polyunsaturated fatty acids.

There have been many studies examining the relationship between the Mediterranean diet and muscle health, including sarcopenia. A systematic review of prospective cohort studies investigating the relationship between diet quality and muscle-related indices among older adults indicated that the Mediterranean diet and Nordic diet are associated with a decreased risk of sarcopenia [26]. In the European Prospective Investigation into Cancer (EPIC)-Norfolk cohort, greater adherence to a Mediterranean diet was significantly associated with higher indices of fat-free mass (FFM) [27]. However, a prospective cohort study of older Chinese men and women found no significant association between Mediterranean Diet adherence and incidence of frailty [28]. A cohort study conducted with older Japanese adults found that a dietary pattern rich in vegetables, fruits, and fish—though not a Mediterranean diet—was inversely associated with a lower risk of disability [29].

Higher adherence to the Mediterranean diet, which is rich in a variety of micronutrients, may play a crucial role in preserving skeletal muscle outcomes. This effect may stem from their potential anti-inflammatory and antioxidant properties or through direct influence on muscle metabolism and physiology [30,31,32]. While individual dietary constituents within the Mediterranean diet contribute significantly to muscle mass and function, the combined interactive and synergistic effects of these components are likely to have a greater impact. However, ethnic differences among populations and potential modifications to the components of the Mediterranean diet may influence these outcomes.

Interestingly, high adherence to the alcohol and side dish pattern, which is one of the Korean food cultures, was associated with an approximately 20% increased probability of both CO and LMM. Side dishes accompanying alcoholic beverages, also called ‘Anju’ in Korean, consist of a variety of foods, including grilled pork belly (samgyeopsal), fried chicken, dried squid, flattened dried fish, grilled seafood, or noodles with soup. Alcohol consumption has been consistently associated with muscle loss or sarcopenia across several studies [33,34]. Previous Korean cross-sectional studies using KNHANES data found that high-risk alcohol drinking was also associated with an increased risk of sarcopenia [35]. Chronic excess alcohol consumption can lead to dysbiosis of the gut microbiota and autophagy-induced hyperammonemia, instigating the upregulation of muscle protein breakdown and downregulation of muscle protein synthesis through the deactivation of IGF-1 and activation of myostatin, AMPK (AMP-activated protein kinase), and REDD1 (regulated in development and DNA damage response 1) [33,36]. Additional mechanisms may involve the secretion of inflammatory cytokines and glucocorticoids [32].

Research findings on whether alcohol causes obesity are somewhat conflicting. However, evidence suggests that alcohol suppresses lipid oxidation in adipose tissue, thereby promoting fat deposition, particularly in the abdominal region [37,38,39]. Rather than directly converting into fat itself, alcohol aggravates the accumulation of fat in the body through various bypass mechanisms, including hindering fat burning, boosting overall food consumption, and increasing fat intake [39]. Research indicates that 70–80% of alcohol is converted into acetate in the liver, after which it enters the bloodstream. The acetate is then transported to muscle and adipose tissues, where it is utilized as an energy source. This process inhibits the breakdown and utilization of body fat, potentially leading to the worsening of obesity [39]. Moreover, it is known that not only calories from alcohol but also calories consumed from side dishes accompanying alcohol contribute to excessive nutritional intake, which has a negative impact on obesity and health [40]. The strong association between the ‘alcohol and side dish pattern’ and CO observed in our study may also be due to the fact that side dishes paired with alcohol primarily consist of oily, fatty, or salty foods. For example, ‘chi-mac’, a Korean term for fried or barbecued chicken paired with beer, and ‘samgyeopsal and soju’, which refer to pork belly and distilled Korean liquor, are typical and popular combinations of alcohol and side dishes [41]. In other words, dietary patterns that combine alcohol consumption with the intake of inherently obesity-inducing foods can significantly contribute to the development of abdominal obesity.

Another prevalent dietary pattern observed in this study was the convenient-based dietary pattern’, characterized by a low consumption of traditional staples, such as cooked rice, and a high consumption of convenience foods and fast food, particularly those containing refined carbohydrates (refined flours or sugars). Although the results were marginally significant, there exists a potential association with LMM, suggesting that the nutritional imbalance resulting from a diet characterized by a reduced intake of protein-based foods, alongside increased consumption of refined carbohydrates, high glycemic index foods, and saturated fats, may contribute to the decline in muscle health. Individuals with normal muscle mass and high hand-grip strength had greater daily energy, protein, and fat intake and a lower percentage of energy from carbohydrates compared to sarcopenic individuals [42]. The consumption of highly processed foods may play a particularly important role, as it is a common feature of low-quality diets, which are also characterized by a low intake of fruits and vegetables [15]. Among older adults, frequent consumption of ultra-processed foods was associated with an increased likelihood of sarcopenia [43] and a greater annual decline in grip strength [44] compared to those who consume such foods less frequently.

Although not directly addressed in the current study, a sedentary lifestyle or physical inactivity is among the most significant factors negatively affecting fat accumulation and skeletal muscle metabolism [15,45]. The association between physical activity, CO, LMM, and SO was previously examined in our earlier study [8]. In that study, we examined the combined effects of energy intake, protein intake, and physical activity on body composition phenotypes in the older adult population of Korea. While the likelihood of CO, LMM, and SO was reduced in the group meeting physical activity recommendations, the combined effects of energy intake and physical activity produced varied outcomes for these body composition phenotypes. As the primary objective of the current study is to explore the relationship between dietary patterns and body composition phenotypes, we have minimized the discussion of physical activity.

The current study has several limitations. Firstly, due to its cross-sectional study design, drawing causal or directional inferences from our findings is challenging. Nevertheless, in order to mitigate the potential for reverse-causation bias, we excluded participants who self-reported severe diseases capable of influencing body composition, as well as those who reported alterations in diet at the time of physical examination. Secondly, the 2008–2012 KNHANES provides daily frequency estimates for 63 food items, although information on portion size was not collected. The KNHANES FFQ is not designed to offer precise data for deriving absolute intake estimates for nutrients or foods; its purpose is to provide a general overview of average intake over a more extended time frame than the 24 h dietary recall method. Previous reports have suggested that a frequency-only FFQ may be sufficient for analyzing diet–disease relationships, as portion size contributes relatively less to the variation in dietary intake compared to variations in consumption frequency [21,46]. Thirdly, we adjusted the gender variable to account for differences in the association between body composition phenotype and dietary patterns, but this adjustment may not fully explain gender differences, especially considering the inherent differences in body composition between genders. Finally, there is a possibility of residual confounding by unmeasured or uncontrolled confounders, such as smoking, menopausal status, and genetic predispositions to obesity.

Even with these limitations, our study provides a more comprehensive perspective on dietary interventions aimed at preventing and managing abdominal obesity, muscle loss, and their combination by using factor analysis to assess overall dietary patterns. This approach emphasizes dietary behaviors and patterns rather than focusing solely on specific nutrients or foods, allowing for a deeper understanding of how people consume foods in combination and how these patterns might affect overall health [9,47]. The Korean dietary practice of consuming foods high in saturated fats and salt alongside alcoholic beverages may adversely impact abdominal fat accumulation and muscle composition in older adults. In contrast, recommendations for a traditional healthy eating pattern that includes grains, vegetables, legumes, fruits, fish, and seaweed could serve as effective and optimal intervention strategies for promoting and maintaining a healthy body composition and supporting skeletal muscle health. Our research provides valuable insights for developing culturally relevant dietary interventions aimed at preventing central obesity and muscle loss in aging populations.

## 5. Conclusions

In this study, the effects of dietary patterns on CO, LMM, and SO in the older adult Korean population were examined. The findings provide valuable evidence that highlights the potential role of specific dietary patterns in the prevention and management of these conditions. These results suggest that dietary intervention strategies should be prioritized based on body composition phenotypes, and tailoring interventions accordingly may enhance the effectiveness of strategies aimed at reducing the risk of sarcopenia and sarcopenic obesity. This research underscores the importance of adopting culturally relevant dietary patterns that promote healthy aging.

## Figures and Tables

**Table 1:** Characteristics of the study population according to central obesity and low muscle mass.

Variables	HC (*n* = 2737)	CO (*n* = 1815)	LMM (*n* = 1389)	SO (*n* = 179)	*p*-Value
Age, years (Mean ± SD)	62.8 ± 8.8	63.9 ± 8.4	65.8 ± 9.4	68.8 ± 8.0	<0.001
50−64	1591 (58.1)	968 (53.3)	620 (44.6)	54 (30.2)	<0.001
65−74	826 (30.2)	634 (34.9)	449 (32.3)	74 (41.3)	
≥75	320 (11.7)	213 (11.7)	320 (23.0)	51 (28.5)	
Sex					<0.001
Male	1149 (42.0)	655 (36.1)	774 (55.7)	81 (45.3)	
Female	1588 (58.0)	1160 (63.9)	615 (44.3)	98 (54.8)	
Place of residence					0.744
Urban area (Dong)	1707 (62.4)	1118 (61.6)	872 (62.8)	117 (65.4)	
Rural area (Eup/Myeon)	1030 (37.6)	697 (38.4)	517 (37.2)	62 (34.6)	
Marital status					0.002
Married	2146 (78.6)	1356 (75.0)	1086 (78.4)	125 (70.2)	
Separated, divorced, or widowed	570 (20.9)	446 (24.7)	286 (20.6)	51 (28.7)	
Never married	15 (0.6)	7 (0.4)	14 (1.0)	2 (1.1)	
Educational level					<0.001
Elementary school graduate or less	1420 (52.3)	1089 (60.3)	740 (53.8)	116 (65.5)	
Middle school graduate	475 (17.5)	311 (17.2)	237 (17.2)	27 (15.3)	
High school graduate	549 (20.2)	276 (15.3)	261 (19.0)	24 (13.6)	
College graduate or more	270 (10.0)	129 (7.2)	138 (10.0)	10 (5.7)	
Occupation					<0.001
White-collar worker	155 (5.7)	98 (5.4)	82 (6.0)	8 (4.5)	
Pink-collar worker	252 (9.3)	188 (10.4)	97 (7.1)	14 (7.9)	
Blue-collar worker	1180 (43.5)	676 (37.5)	519 (37.8)	40 (22.6)	
Unemployed	1123 (41.4)	842 (46.7)	675 (49.2)	115 (65.0)	
Household income					<0.001
Quartile 1 (lowest)	851 (31.5)	639 (35.7)	544 (39.7)	63 (36.0)	
Quartile 2	695 (25.7)	486 (27.1)	366 (26.7)	62 (35.4)	
Quartile 3	611 (22.6)	338 (18.9)	229 (16.7)	32 (18.3)	
Quartile 4 (highest)	549 (20.3)	328 (18.3)	232 (16.9)	18 (10.3)	
Level of moderate-to-vigorous-intensity physical activity					<0.001
No physical activity	1269 (46.7)	980 (54.3)	833 (60.5)	122 (69.3)	
Insufficient level of physical activity	260 (9.6)	182 (10.1)	103 (7.5)	19 (10.8)	
Recommended level of physical activity	1190 (43.8)	643 (35.6)	440 (32.0)	35 (19.9)	
BMI, m/kg^2^ (Mean ± SD)	23.1 ± 2.0	26.8 ± 2.4	20.7 ± 2.0	24.4 ± 1.8	<0.001
Hypertension					<0.001
No	1413 (51.6)	620 (34.2)	723 (52.0)	53 (29.6)	
Yes	1066 (38.9)	1074 (59.2)	555 (40.0)	117 (65.4)	
Unknown	258 (9.4)	121 (6.7)	111 (8.0)	9 (5.0)	
Diabetes mellitus					<0.001
No	2345 (85.7)	1356 (74.7)	1108 (79.8)	125 (69.8)	
Yes	247 (9.0)	350 (19.3)	162 (11.7)	39 (21.8)	
Unknown	145 (5.3)	109 (6.0)	119 (8.6)	15 (8.4)	

HC, healthy control; CO, central obesity; LMM, low muscle mass; SO, sarcopenic obesity; SD, standard deviation. Data are n (%) or Mean ± SD.

**Table 2:** Factor loading matrix for the three dietary patterns identified by factor analysis.

	Factor 1	Factor 2	Factor 3
Food or Food Group	Healthy Dietary Pattern	Convenience-Based Dietary Pattern	Alcohol and Side Dish Pattern
Rice	0.24	**−0.53**	−0.06
Other gains	**0.51**	−0.25	−0.31
Noodles	−0.03	0.16	**0.47**
Bread, sweets, and confectionery	0.10	**0.56**	0.01
Rice cakes	0.24	**0.46**	−0.16
Legumes and legume products	**0.57**	−0.23	−0.26
Potatoes and sweet potatoes	**0.42**	0.19	−0.02
Meats (beef, pork, and chicken)	0.38	0.34	**0.40**
Eggs	0.36	0.25	0.22
Processed meats	0.21	0.39	0.31
Fish and seafood	**0.61**	0.17	0.27
Salted fish and seafood	0.16	−0.15	**0.43**
Vegetables	**0.65**	−0.12	0.24
Mushrooms	**0.51**	0.17	0.22
Seaweed	**0.54**	0.15	0.16
Fruits	**0.60**	0.35	−0.06
Dairy products	0.36	**0.45**	−0.06
Soda	−0.03	0.27	0.24
Coffee and tea	0.11	0.16	0.39
Alcoholic beverages	−0.04	−0.18	**0.60**
Fast foods (hamburger and pizza)	0.11	**0.48**	0.11
Fried foods	0.12	0.36	0.32
Variance explained by each factor	3.10	2.26	1.79

Values are factor loading; absolute values ≥0.40 are in bold. A positive loading indicates that a food group is positively associated with the factor; a negative loading denotes an inverse association.

**Table 3:** Distribution of participants according to dietary pattern tertiles.

Variables	HC	CO	LMM	SO	*p*-Value
Healthy dietary pattern					0.002
Tertile 1	843 (30.8)	637 (35.1)	487 (35.1)	73 (40.8)	
Tertile 2	927 (33.9)	589 (32.5)	463 (33.3)	61 (34.1)	
Tertile 3	967 (35.3)	589 (32.5)	439 (31.6)	45 (25.1)	
Convenience-based dietary pattern					0.238
Tertile 1	904 (33.0)	584 (32.2)	493 (35.5)	59 (33.0)	
Tertile 2	896 (32.7)	637 (35.1)	441 (31.8)	66 (36.9)	
Tertile 3	937 (34.2)	594 (32.7)	455 (32.8)	54 (30.2)	
Alcohol and side dish pattern					0.005
Tertile 1	915 (33.4)	639 (35.2)	414 (29.8)	72 (40.2)	
Tertile 2	938 (34.3)	580 (32.0)	466 (33.6)	56 (31.3)	
Tertile 3	884 (32.3)	596 (32.8)	509 (36.7)	51 (28.5)	

HC, healthy control; CO, central obesity; LMM, low muscle mass; SO, sarcopenic obesity. Data are n (%).

**Table 4:** Multivariate association between body composition phenotype and dietary patterns.

		Crude Model			Multivariate Model ^1^	
Variables	CO OR (95% CI)	LMM OR (95% CI)	SO OR (95% CI)	CO OR (95% CI)	LMM OR (95% CI)	SO OR (95% CI)
Healthy dietary pattern						
Tertile 1	1.00	1.00	1.00	1.00	1.00	1.00
Tertile 2	0.84 (0.73–0.97)	0.87 (0.74–1.01)	0.76 (0.53–1.08)	0.87 (0.74–1.01)	0.91 (0.77–1.07)	0.76 (0.52–1.09)
Tertile 3	0.81 (0.70–0.93) *	0.79 (0.67–0.92) *	0.54 (0.37–0.79) **	0.88 (0.75–1.04)	0.90 (0.76–1.07)	0.61 (0.40–0.92)
P for trend	0.004	0.003	0.002	0.141	0.259	0.018
Convenience-based dietary pattern						
Tertile 1	1.00	1.00	1.00	1.00	1.00	1.00
Tertile 2	1.10 (0.95–1.27)	0.90 (0.77–1.06)	1.13 (0.79–1.62)	1.21 (1.04–1.41) *	1.03 (0.88–1.22)	1.40 (0.96–2.04)
Tertile 3	0.98 (0.85–1.14)	0.89 (0.76–1.04)	0.88 (0.60–1.29)	1.11 (0.95–1.31)	1.18 (0.99–1.41) *	1.31 (0.87–1.98)
P for trend	0.642	0.169	0.443	0.292	0.056	0.232
Alcohol and side dish pattern						
Tertile 1	1.00	1.00	1.00	1.00	1.00	1.00
Tertile 2	0.89 (0.77–1.02)	1.10 (0.94–1.29)	0.76 (0.53–1.09)	0.96 (0.83–1.12) *	1.05 (0.89–1.25)	0.86 (0.59–1.25)
Tertile 3	0.97 (0.84–1.12)	1.27 (1.09–1.49) **	0.73 (0.51–1.06)	1.22 (1.03–1.44) **	1.21 (1.01–1.45) *	0.98 (0.64–1.50)
P for trend	0.746	0.003	0.113	0.016	0.043	0.959

HC, healthy control; CO, central obesity; LMM, low muscle mass; SO, sarcopenic obesity; OR, odds ratio; CI, confidence interval. ^1^ Adjusted for age, sex, place of residence, marital status, education level, occupation, household income, physical activity, hypertension, and diabetes mellitus. * *p* < 0.05; ** *p* < 0.01.

## Data Availability

The data of the current study are available from the KDCA (https://knhanes.kdca.go.kr/knhanes/eng/index.do (accessed on 28 January 2022)) on reasonable request.

## Supplementary Materials

The following supporting information can be downloaded at: https://www.mdpi.com/article/10.3390/nu16213583/s1, Table S1: Food groups and food items from the FFQ in the study.
