# Supplementary material for: Dietary Patterns Associated with Body-Composition Phenotype in a Middle-Aged and Elderly Population: A Population-Based Cross-Sectional Study
Source: Nutrients. 2024 Oct 22;16(21):3583. doi: 10.3390/nu16213583 (PMC11547401; doi:10.3390/nu16213583)
Supplement: Supplementary file 1 [file nutrients-16-03583-s001.zip › nutrients-3217185-supplementary.pdf]

Supplementary Table S1. Food groups and food items from the FFQ in the study

**Food groups**	**Sixty-three food items from the FFQ**
Rice	Rice
Other gains	Other gains
Noodles	Instant noodles; noodles (Naengmyeon, Udon, and Kalguksu)
Bread, sweets, and confectionery	Breads (including all breads); confectionery
Rice cakes	Rice cakes (including Tteokbokki and Tteokguk)
Legumes and legume products	Legumes; Tofu (including soft tofu); soy milk
Potatoes and sweet potatoes	Potatoes; sweet potatoes
Meat (beef, pork, and chicken)	Beef; chicken; pork
Eggs	Eggs
Processed meat	Ham, bacon, and sausage (including hot dogs); Korean fish cakes (Odeng)
Fish and seafood	Mackerel; tuna; croaker; pollack; anchovy; squid (including dried squid); shellfish
Salted fish and seafood	Salted fish and seafood (Jeotgal)
Vegetables	Korean cabbage (including Kimchi); Korean radish; greens of Korean radishes (Mucheong); bean sprouts; spinach; cucumbers; red pepper; carrot; zucchini; cabbage; tomatoes (including juice)
Mushrooms	Mushrooms
Seaweed	Sea mustard (Mi-yeog); laver (Gim)
Fruits	Tangerines (including kumquats, juice, and canned food); persimmons and dried persimmons; Korean pear; Watermelon; Korean melon; strawberry; grapes (including juice and canned food); peach (including juice and canned food); apple (including juice); banana; orange (including juice)
Dairy products	Milk; yogurt (including liquid and semi-solid); ice cream;
Soda	Soda (including Cola, Cider, and Fanta)
Coffee and tea	Coffee; green tea
Alcohol	Beer; Soju; traditional Korean rice liquor (Makgeolli)
Fast food (hamburgers and pizza)	Hamburger; pizza
Fried foods	Fried foods
